# Evaluation of in-house, haptic assisted surgical planning for virtual reduction of complex mandibular fractures

**DOI:** 10.1007/s11548-021-02353-w

**Published:** 2021-04-27

**Authors:** Johanna Nilsson, Fredrik Nysjö, Ingela Nyström, Johan Kämpe, Andreas Thor

**Affiliations:** 1 Oral and Maxillofacial Surgery, Zealand University Hospital, Roskilde, Denmark; 2 Plastic and Oral and Maxillofacial Surgery, Department of Surgical Sciences, Uppsala University, Uppsala, Sweden; 3 Department of Information Technology, Centre for Image Analysis, Uppsala University, Uppsala, Sweden

**Keywords:** Virtual surgery planning, Haptics, Cranio-maxillofacial surgery

## Abstract

**Purpose:**

The management of complex mandible fractures, i.e. severely comminuted or fractures of edentulous/atrophic mandibles, can be challenging. This is due to the three-dimensional loss of bone, which limits the possibility for accurate anatomic reduction. Virtual surgery planning (VSP) can provide improved accuracy and shorter operating times, but is often not employed for trauma cases because of time constraints and complex user interfaces limited to two-dimensional interaction with three-dimensional data.

**Methods:**

In this study, we evaluate the accuracy, precision, and time efficiency of the haptic assisted surgery planning system (HASP), an in-house VSP system that supports stereo graphics, six degrees-of-freedom input, and haptics to improve the surgical planning. Three operators performed planning in HASP on computed tomography (CT) and cone beam computed tomography (CBCT) images of a plastic skull model and on twelve retrospective cases with complex mandible fractures.

**Results:**

The results show an accuracy and reproducibility of less than 2 mm when using HASP for virtual fracture reduction, with an average planning time of 15 min including time for segmentation in the software BoneSplit.

**Conclusion:**

This study presents an in-house haptic assisted planning tool for cranio-maxillofacial surgery with high usability that can be used for preoperative planning and evaluation of complex mandible fractures.

## Introduction

Mandible fractures are one of the most common fracture types in the facial area, often resulting from traffic accidents, sports injuries, or assault [1]. This can be explained by the mandible’s prominent anatomical location in the face. The mandible is an important aesthetic and functional structure, which contributes to the frame of the face and acts as a protective structure against trauma to vital areas of the head. The decision making for treatment of mandible fractures is dependent on the location, the stability, the degree of displacement, and the influence on the occlusion [1–4]. For most mandible fractures, the treatment strategy is relatively straightforward, but when it comes to more complex fractures with multiple segments or an edentulous or atrophic mandible, re-establishment of the three-dimensional (3D) shape can be challenging [5]. Virtual surgical planning (VSP) in the field of cranio-maxillofacial surgery (CMF) has the potential to make post-operative outcomes more accurate, reduce surgical time, and improve the communication between the patient and the clinical team [6]. VSP already plays an important role in the field of orthognathic surgery and reconstructive surgery [6–9]. There is also potential that VSP can be essential in diagnostics and treatment of complex fractures of the mandible [10, 11].

**Fig. 1:**
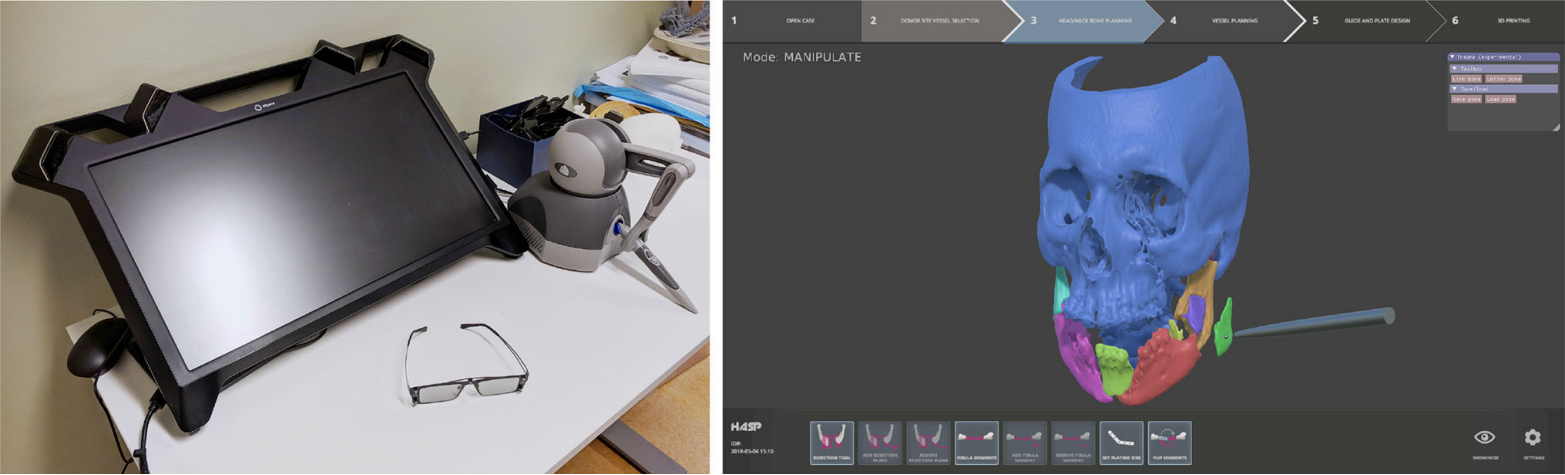
HASP system: (left) hardware including haptic device and head-tracked stereo glasses; (right) planning software displaying the graphical user interface and a visualization of a trauma case

Commercially available VSP systems (Brainlab [12]; Materialise [13]; Planmeca [14]) often involve the interaction with a computer engineer. The patient information (DICOM data from a CT or CBCT scan) is uploaded, and the virtual plan and optional surgical guides and plates are created in close collaboration between the engineer and the surgeon. There are several disadvantages with this set-up. Firstly, the workflow with correspondence and approval can take several days, which is not appropriate in the field of trauma. Secondly, the commercially available VSP systems in CMF are primarily relying on complex 2D graphical user interfaces and require the user to visualize and interact with the 3D anatomical structures from a 2D view. While a trained user can work efficiently in such systems, they would take too much time and effort for most surgeons to learn.

Haptic technology recreates the sense of touch by applying a force or vibration back to the user from a simulation in a virtual environment. It is used to improve the realism in simulators and is already available in training for specific surgical procedures [15]. Haptics have the potential to simplify preoperative planning by giving the surgeon virtual tools that are similar to the procedures in the operating theatre, namely the sensation of the fit of two bone fragments or the dental occlusion [16].

At the Centre for Image Analysis at Uppsala University, a tool for virtual reduction of mandible fractures has been developed [17, 18]. Haptic assisted surgical planning (HASP) is an intuitive, in-house solution for CMF surgery planning, designed for the surgeons to perform all steps in both planning and evaluation. In [17], the authors only show proof of concept of using HASP for trauma cases, using a practice case and an evaluation case. In [18], the system was evaluated for another type of planning (reconstructive fibula flaps).

HASP uses stereo graphics, six degrees-of-freedom (DOF) input, and haptic feedback to improve the surgical planning by allowing easier interaction with 3D data, as shown in Fig. 1. The surgeon manipulates virtual bone fragments in 3D through a haptic device that provides 6DOF input and 3DOF force feedback. The haptic simulation computes a collision response when bone fragments collide, allowing the surgeon to assess the fit between bone fragments and to test whether the occlusion is correct. The haptic simulation also supports in avoiding interpenetration of fragments that may be impossible to detect visually. By wearing head-tracked stereo glasses, the surgeon also receives correct depth perception and the possibility to look around in the virtual scene. Further details about the HASP system are provided in Olsson et al. [17].

The aim of this study was to describe a new workflow for virtual reduction of complex mandible fractures, and to investigate the accuracy and precision of the virtual tool HASP on a larger number of retrospective cases that was used in the previous study [17]. Here, and in the rest of the paper, we will define accuracy as the difference in shape (according to different metrics, for example, overlap) between a virtual reduction and a reference model (the intact mandible), and precision as the difference in shape between repeated reductions.

## Materials and methods

### Evaluation of HASP on simulated bilateral fracture on a plastic skull model

**Fig. 2:**
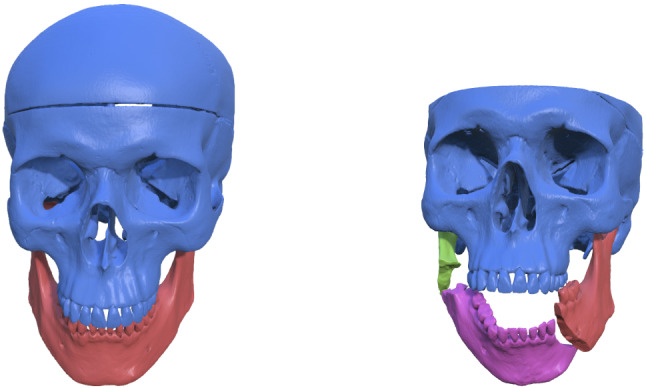
The plastic skull model with the simulated fracture position

**Fig. 3:**
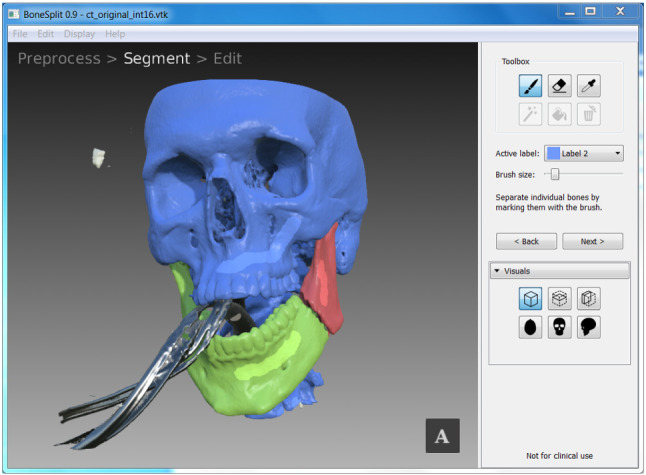
Segmentation in BoneSplit

A standard, commercially available plastic skull (SOMSO, Coburg, Germany) with detailed dental surfaces was used for the evaluation. Image acquisition with CT (SIEMENS Somatom definition flash, pitch 0.8, mas 25, kv 100 and 0.75 mm slices) of the plastic skull model in a maximum intercuspation position (MIP) was obtained. The model was used as a reference model to evaluate the accuracy and precision of the software. A bilateral fracture was created on the plastic model using an oscillating saw. The bone fragments were moved from their original position into a simulated “fracture position”, as shown in Fig. 2. Thereafter, a new CT scan was obtained. The CT data were first imported in DICOM format into BoneSplit, an interactive segmentation tool that uses a 3D texture–painting interface for bone separation [19]. The semi-automatic segmentation in BoneSplit enables quick separation of individual bone fragments after an initial threshold value for bone has been selected, by using a graph-based segmentation method to automatically compute a label image from seeds that the user paints on the surface of the bone, as shown in Fig. 3. Segmentation of the individual bone parts (cranium and the fractured parts of the mandible) was performed by one of the authors, and the time spent for each case was measured. After segmentation, the segmented data were imported into HASP for virtual reduction planning. Three observers (U1, U2, and U3) performed the virtual reduction twice to evaluate the accuracy and the reproducibility. For each virtual reduction, stereolithography (STL) models and poses of the bone fragments were saved. The time spent for the planning for each case was measured.

During the study, it was observed that the virtual reductions by the observers were narrower than the intact mandible. In order to investigate this, the physical model of the artificially fractured plastic mandible was separately assembled with glue by a surgeon (one of the authors), and scanned with a CT scanner.

The accuracy of the planning tool was evaluated by superimposing the virtual planned mandible on the intact plastic skull and measuring the overlap and deviation between the two objects.

**Fig. 4:**
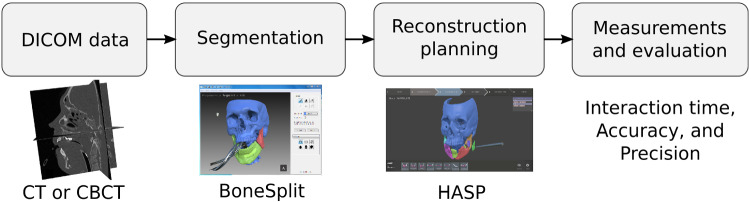
Overview of the workflow in the study: (left) CT or CBCT acquisition; (middle left) segmentation of the different bone parts in BoneSplit; (middle right) virtual reduction in HASP; (right) evaluation of interaction times and measurements of the accuracy and precision reproducibility by superimposing and measuring deviation

### Evaluation of HASP on retrospective data of patients with complex bilateral mandible fractures

A search for bilateral mandible fractures in the patient database at the department of Oral and Maxillofacial surgery at Uppsala University between 2015 and 2017 was made. Inclusion criteria were a mandible fracture with at least two displaced fractures or more with existing pre- and post-operative CT/CBCT with a slice thickness of less than 1 mm. The CT or CBCT data were imported into BoneSplit, and after segmentation, it was imported into HASP, as described for the plastic skull. Since BoneSplit was originally developed for segmentation of CT data, the threshold value for the CBCT datasets had to be adjusted manually for these cases, to include as much bone tissue as possible and to compensate for the lower image contrast that CBCT provides. The same three observers (U1, U2, and U3) performed the planning with two repetitions each. Evaluation of the reduction planning was made by measuring the inter- and intra-observer variability for the twelve cases.

## Measurements

To measure volume overlap between reconstructions, the software Binvox [20] was used to first binary voxelize the STL models of each reconstruction into a common volume, such that a single volume per user and trial was obtained. The software Convert3D [21] was then used to compute Dice coefficients between pairs of volumes. Fragment pose mean angular difference between reconstructions was computed in a Python script from exported fragment pose matrices and defined as the mean absolute angle of the quaternion differences. Surface-to-surface two-sided Hausdorff distance and mean absolute distance (MAD) were finally measured using the software MeshLab; more details on how these measurements are computed can be found in Cignoni et al. [22].

**Fig. 5:**
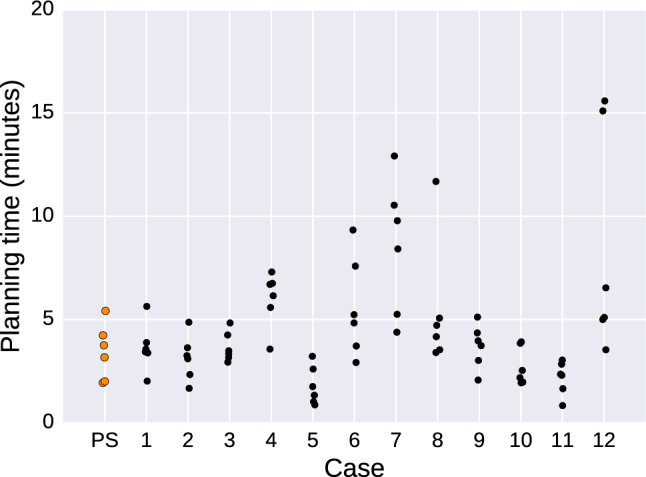
Overview of the time spent for each case in HASP. The orange data points represent the plastic skull (PS)

## Results

The workflow for the planning and evaluation is presented in Fig. 4. The mean time for segmentation was 9.89 (3.55) min. The mean planning interaction time was 4.49 (2.96) min. An overview of time spent for each case is presented in Fig. 5.

### Evaluation of HASP on simulated bilateral fracture on a plastic skull model

**Fig. 6:**
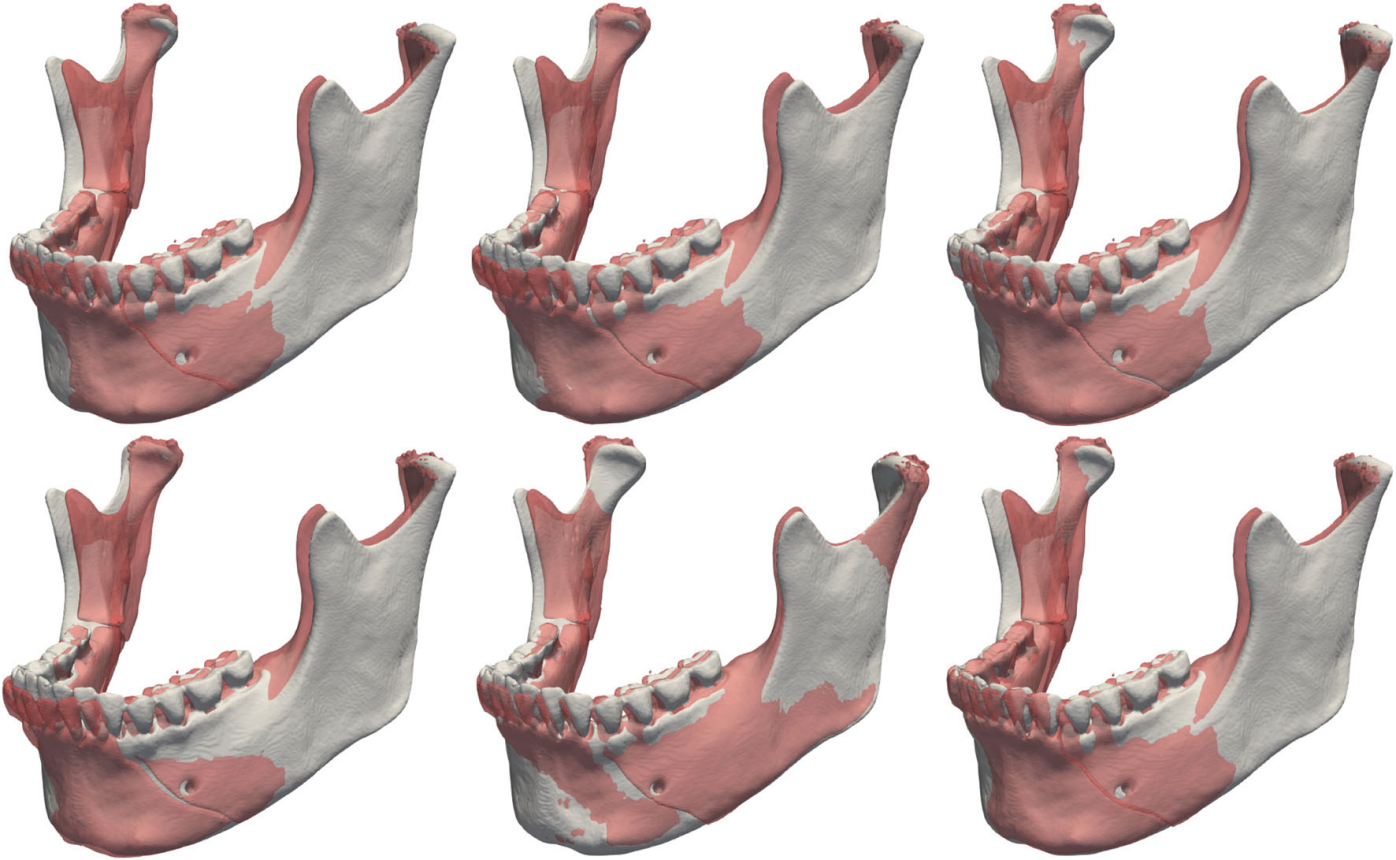
Visualization of the deviation between the intact plastic model (grey) and the position of the virtual reductions (red) performed on the fractured model. The top row shows trial T1 for users U1, U2, and U3 (in order left to right), whereas the bottom row shows trial T2 for the same users (in the same order)

Table 1 shows the accuracy for the planning method, describing the mean absolute distance (MAD), maximum distance (Hausdorff), and the volume overlap (Dice) between the intact plastic skull and the repeated virtual reductions. Table 1 also shows the accuracy between the glued plastic skull and the virtual reductions. Figure 6 shows a visualization of the deviation between the intact plastic model (grey) and the position of the virtual reductions (red) performed on the fractured model.

### Evaluation of HASP on retrospective data of patients with complex bilateral mandible fractures

**Fig. 7:**
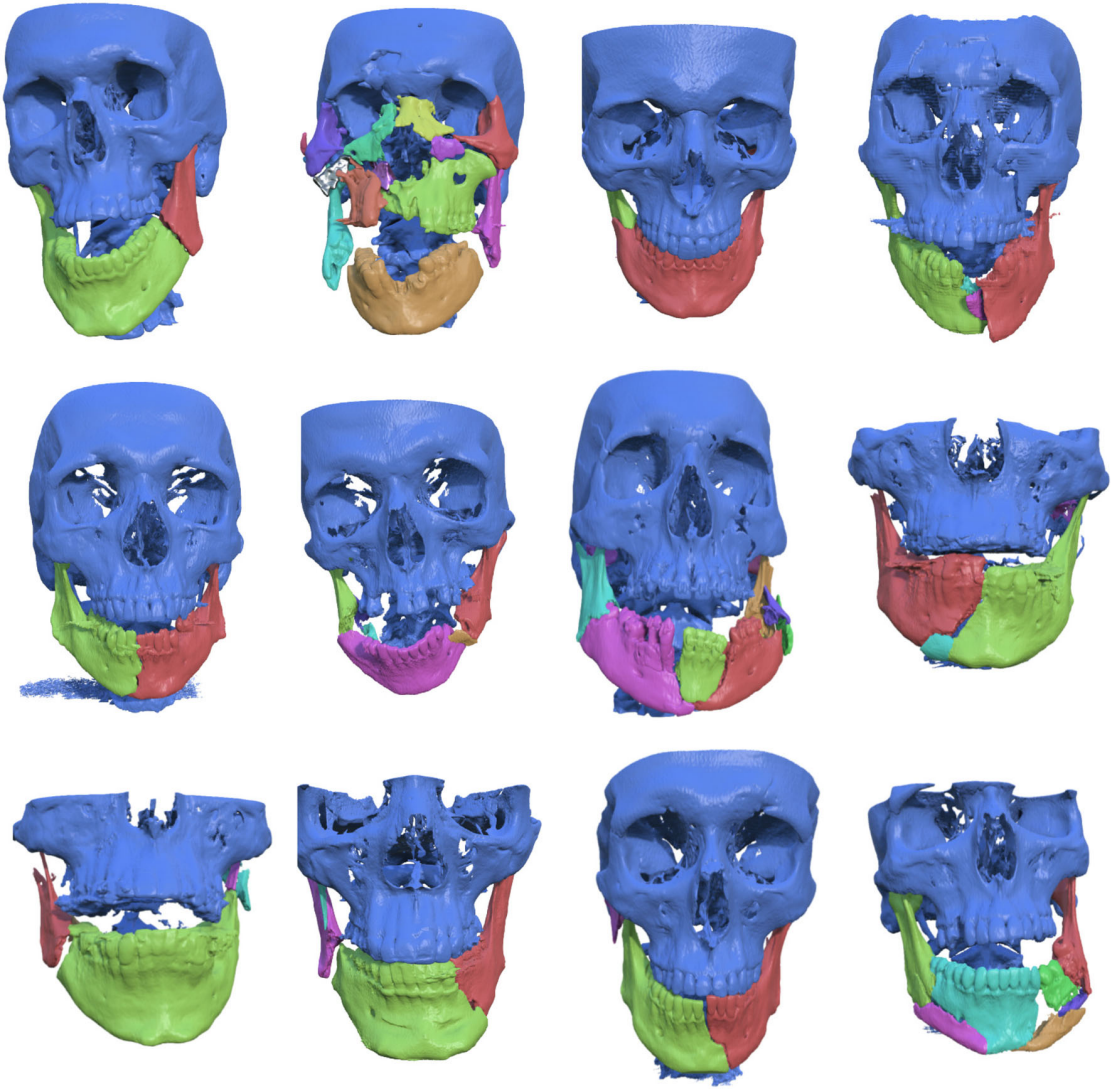
From top left to bottom right (in row-wise order): included retrospective cases 1–12 with complex mandible fractures, after segmentation

**Fig. 8:**
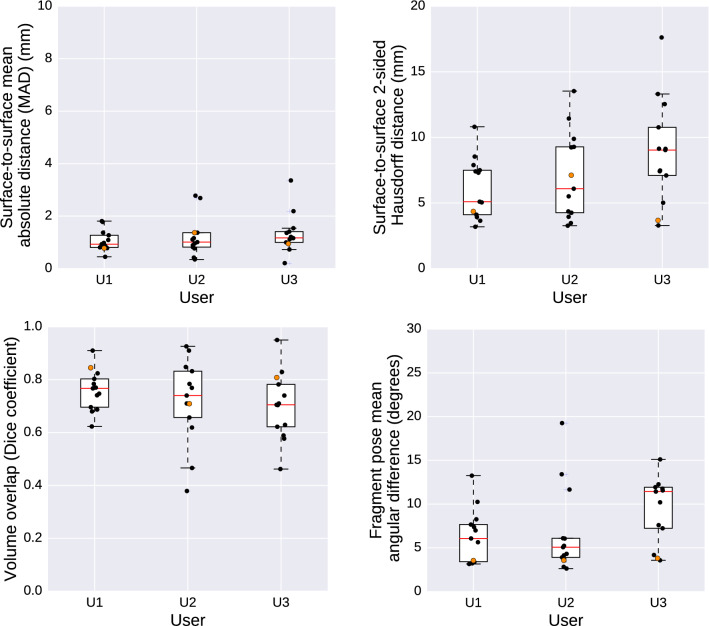
Intra-observer variability. The measurements from the plastic skull model are marked with orange data points

**Fig. 9:**
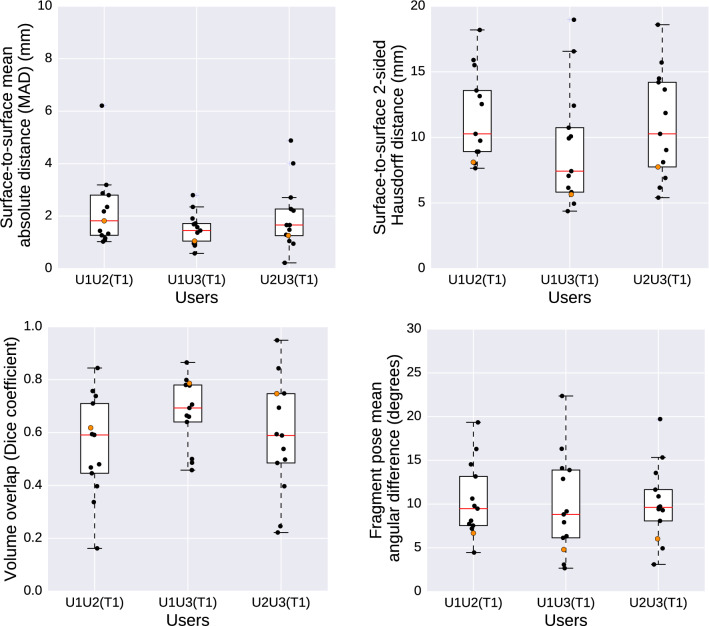
Inter-observer variability. The measurements from the plastic skull model are marked with orange data points

**Fig. 10:**
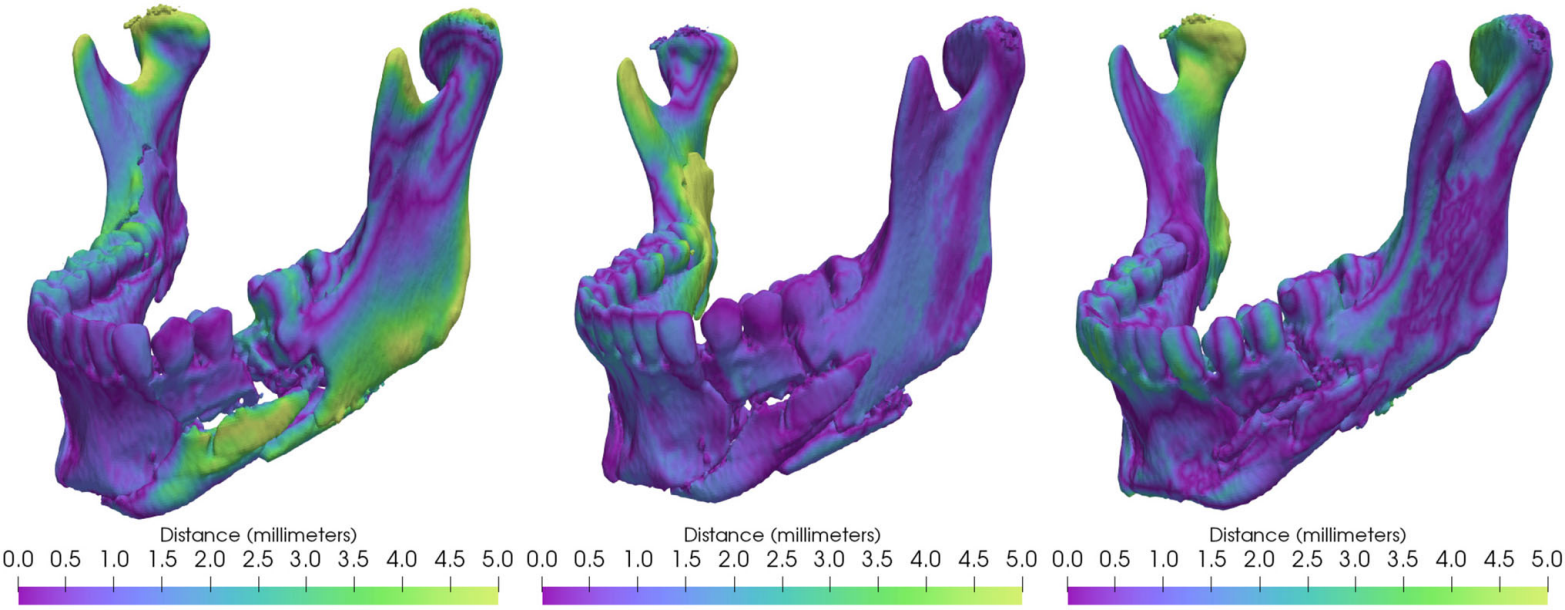
Intra-operator absolute distance presented with a colour coded distance map. A large absolute distance indicates a large deviation between the repeated virtual reductions T1 and T2 by the same user, when the mandibles were aligned by superimposing the skulls

The included mandible fracture cases after segmentation are visualized and presented in Fig. 7. Figure 8 shows the intra-observer variability, described in mean absolute distance (MAD), maximum distance (Hausdorff), the volume overlap (Dice), and the angular difference. Figure 9 shows the inter-observer variability described in mean absolute distance (MAD), maximum distance (Hausdorff), the volume overlap (Dice), and the angular difference. The intra-operator precision for one case (number 12) is visualized with a colour coded distance map in Fig. 10.

## Discussion

The present study describes the workflow for using an in-house VSP system including haptics for complex mandible fractures. This novel planning set-up was shown to be time-efficient, user-friendly, and with high precision and fairly low accuracy.

A limitation of this study was the use of an artificial plastic skull to evaluate the accuracy. A cadaver skull had probably been more appropriate to use, to also present the challenges with segmentation of different types of tissue, i.e. soft and hard tissue. Furthermore, it might have been more suitable to randomise the patient cases, so that the users would not perform the same case two times in a row. The instant repetition of the case could have resulted in bias for the second planning session due to the learning curve and the memory of the previous session.

A further limitation of the study is the lack of comparison with others available planning systems. The reason for this is the set-up of those systems. As described in the article by Thor [10], commercially available planning systems can be used with high accuracy and precision, but the clear downside is the time required for the planning; the time from planning until the patient-specific implant (PSI) was available in the clinic was approximately two weeks. In the patient case described by Thor, this planning set-up was a viable option since it was a revision surgery for failed fracture. This ability to wait for the planning and manufacturing of the implant is rarely an option when it comes to typical, more time-critical trauma cases. An in-house system could reduce the planning time and would also be available outside normal working hours.

The accuracy when measured as mean absolute distance was shown to be less than 2 mm (average 1.65 mm) when comparing the virtual reductions against the intact plastic skull, and less than 1.1 mm (average 0.93 mm) for the same comparison against the glued plastic skull (Table 1). The average intra-operator and inter-operator precision when measured as mean absolute distance were 1.10 mm and 1.65 mm (Figs. 8, 9). It is important to interpret these results with caution, since a deviation of 2 mm may imply like a significant issue in the clinical situation, but may not necessarily have a large impact. Firstly, a minimal rotation at the fracture site will propagate in a large deviation at the peripheral anatomical structures, leading to a high mean absolute distance between two objects. In this case, the clinical impact at the fracture site remains low, something that is not reflected in the numbers. Secondly, when evaluating the reproducibility (intra- and inter-observer variability) for the retrospective cases, it is not possible to know the original shape of the mandible. Hence, two virtual reductions can only be compared to each other but not to the original state. Moreover, a small move or rotation of a mandible in one virtual reduction compared to another can result in significant measured deviations even though the two virtual reductions may have exactly the same shape. This is because the measurements were made by superimposing the skull and then calculating the deviations between two virtual planned mandibles.

Limitations of the current HASP system include limited resolution in the number of contact points in the haptic simulation, and the use of a haptic device only supporting 3DOF force output (no torque output). Improving the performance of the haptic simulation could increase the haptic fidelity of the system and thereby make it easier for users to tell when bone fragments fit together. It would also make it more interesting to compare how an user performs in our system with and without the haptic feedback enabled. Because our collision simulation is based on a virtual coupling to prevent interpenetration between fragments, disabling haptic feedback in the current implementation of HASP would likely mainly affect the user interaction time (not the accuracy or precision). Regarding segmentation, possible future work involves exploring methods for hole filling to address the problem with hollow mandibles affecting the volume overlap score in the measurements. In the comparisons between virtual reductions against the glued plastic skull (where both mandibles were solid), the volume overlap scores in Table 1 can be seen to have values above the upper quartile in the corresponding boxplots of Figs. 8 and 9.

Accurate fracture reduction is important for re-establishing the original anatomy and thereby the function. Inadequate reduction and fixation can result in post-operative complications including infection, malunion/nonunion, malocclusion, or mandible flaring with a posterior cross-bite [23]. For the individual patient with a complex mandible fracture, accurate virtual preoperative planning has the potential to shorten the time spent in surgery and to improve the aesthetics and function. As an example, a big challenge in CMF surgery is treatment of severely atrophic and edentulous mandibles. Resorption of the alveolar process is a natural, progressive event after loss of teeth. The surgical reduction of the fracture parts is significantly more difficult due to the lack of 3D landmarks and the lack of occlusion as guidance. VSP, including pre-contouring of the plate or creating a patient specific plate from an exact model of the patient’s mandible, could lead to a more accurate result and a decreased surgical time. Patients with atrophic and edentulous mandibles are mainly seen in the geriatric population, which is a patient group often seen with medical comorbidities where the total time in general anaesthesia is of high importance for the overall morbidity [24].

Clinical studies using VSP for mandibular fractures are rare. Li [25] presented a study, comparing VSP with conventional surgery for complex mandible fractures. The VSP was carried out through a web session, but the physical models were printed at the clinic, reducing the preoperative time that normally includes manufacturing and shipping. The results from the study showed that VSP decreased the total operative time to 54 (3.5) min versus 78 (4.0) min and that the intraoperative bleeding was less than in the control group. They suggested that pre-shaping of the reconstruction plate makes the surgery faster and more accurate, especially for the edentulous patient without a normal occlusion as guidance. Repetitive bending of the plates could also lead to a weakness in the plate and thereby increase the risk for fatigue break, something that can be avoided using VSP.

VSP in the field of trauma surgery needs to be fast and user-friendly. Current commercially available CMF planning systems are based on the collaboration with a computer engineer to perform all the steps with guidance of the surgeon. In both trauma and reconstruction for oncological reasons, the available time for planning is limited, which can cause problems in the traditional set-up with existing planning systems. VSP for trauma may not yet be suitable to be used as standard, but may evolve to be a great advantage in complex cases and in patients with an atrophic and/or edentulous mandible. In those cases, there is a need for a load-bearing osteosynthesis, which often entails a long reconstruction plate. Accurately fitting a long reconstruction plate can be very time-consuming and is highly determined by the surgeon’s level of experience. The advantages of VSP are presented in the literature and show both high accuracy, shorter time spent in surgery and reduced intraoperative bleeding [10, 25, 26]. Navigation and intraoperative C-arm CT are new technical tools that are used in CMF surgery. They have mainly been used in the treatment of orbit reconstruction but also for ballistic facial injuries, including mandible fractures, as described by Kumpfer et al. [27] Intraoperative CT scans can be helpful to detect errors early in the surgery and decrease the risk for reoperations. We are currently facing a new technical era where the focus has shifted from the surgeon’s subjective assessment to a more structured set-up of pre-operative virtual planning and intra-operative execution, with improvement in efficiency and outcome. The VSP surgeries and the conventional surgeries made by freehand need to be compared in further prospective studies to evaluate potential clinical benefits and thereby justifying the initial additional costs.

**Table 1:** Accuracy data for our planning method, describing the mean absolute distance (MAD), maximum distance (Hausdorff), and the volume overlap (Dice) between the intact plastic skull and the repeated virtual reductions T1 and T2 performed by users U1–U3. The table also shows the accuracy between the glued skull and the same virtual reductions

	MAD	Max	Volume overlap (DICE)
U1T1-INTACT	1.88	8.57	0.63
U1T2-INTACT	1.73	6.87	0.66
U2T1-INTACT	1.76	6.94	0.65
U2T2-INTACT	1.09	4.85	0.79
U3T1-INTACT	1.7	8.05	0.66
U3T2-INTACT	1.73	7.45	0.66
Mean Min/max	1.65 [1.09, 1.88]	7.12 [4.85, 8.57]	0.67 [0.63, 0.79]
U1T1-GLUED	1.05	4.54	0.8
U1T2-GLUED	0.93	4.37	0.82
U2T1-GLUED	1.03	4.15	0.81
U2T2-GLUED	0.8	3.41	0.85
U3T1-GLUED	0.82	3.62	0.85
U3T2-GLUED	0.94	3.47	0.82
Mean Minmax	0.93 [0.8, 1.05]	3.93 [3.41, 4.54]	0.83 [0.8, 0.85]

The eventual goal of HASP is to provide the surgeon the ability to apply the virtual surgical planning in a fast, intuitive way, to facilitate the different steps through the surgery, including plate choice and pre-operative modification of the plate. This would potentially simplify the reduction of the fracture and the placement of the plate and possibly lead to a faster, more accurate surgery with less post-operative complications. A well-designed prospective clinical study is the next step to assess these claims.

In this study, we present an in-house haptic assisted planning tool with high usability that can be used for pre-operative planning and evaluation of complex mandible fractures. The time for the virtual planning takes around 15 min including segmentation. The intra-observer precision was 1.10 mm, and the inter-observer precision was 1.90 mm, whereas the accuracy was 1.65 mm. The planning system showed high precision and fairly low accuracy.
